# Monocyte Expressed Macromolecular C1 and C1q Receptors as Molecular Sensors of Danger: Implications in SLE

**DOI:** 10.3389/fimmu.2014.00278

**Published:** 2014-06-26

**Authors:** Berhane Ghebrehiwet, Kinga K. Hosszu, Alisa Valentino, Yan Ji, Ellinor I. B. Peerschke

**Affiliations:** ^1^Departments of Medicine and Pathology, Stony Brook University, Stony Brook, NY, USA; ^2^Departments of Laboratory Medicine, Memorial Sloan-Kettering Cancer Center, and Laboratory Medicine and Pathology, Weill-Cornell Medical College, New York, NY, USA

**Keywords:** c1q, DC and C1, monocyte C1, C1q in SLE, C1q in autoimmunity, C1q and C1q receptors

## Abstract

The ability of circulating blood monocytes to express C1q receptors (cC1qR and gC1qR) as well as to synthesize and secrete the classical pathway proteins C1q, C1r, and C1s and their regulator, C1-INH is very well established. What is intriguing, however, is that, in addition to secretion of the individual C1 proteins monocytes are also able to display macromolecular C1 on their surface in a manner that is stable and functional. The cell surface C1 complex is presumably formed by a Ca^2+^-dependent association of the C1r_2_⋅C1s_2_ tetramer to C1q, which in turn is anchored via a membrane-binding domain located in the N-terminus of its A-chain as shown previously. Monocytes, which circulate in the blood for 1–3 days before they move into tissues throughout the body, not only serve as precursors of macrophages and dendritic cells (DCs), but also fulfill three main functions in the immune system: phagocytosis, antigen presentation, and cytokine production. Since the globular heads of C1q within the membrane associated C1 are displayed outwardly, we hypothesize that their main function – especially in circulating monocytes – is to recognize and capture circulating immune complexes or pathogen-associated molecular patterns in the blood. This in turn may give crucial signal, which drives the monocytes to migrate into tissues, differentiate into macrophages or DCs, and initiate the process of antigen elimination. Unoccupied C1q on the other hand may serve to keep monocytes in a pre-dendritic phenotype by silencing key molecular players thus ensuring that unwarranted DC-driven immune response does not occur. In this paper, we will discuss the role of monocyte/DC-associated C1q receptors, macromolecular C1 as well as secreted C1q in both innate and acquired immune responses.

## Introduction

Monocytes serve a critical role in adaptive and innate immunity not only by serving as precursors of macrophages and myeloid dendritic cells (DCs), but also by their function in phagocytosis, antigen processing, and presentation, as well as secretion of pro- and anti-inflammatory cytokines. Monocytes are produced by the bone marrow from hematopoietic stem cell precursors called monoblasts, and circulate in the bloodstream for about 3 days before they migrate into tissues throughout the body where they develop into different types of macrophages and DCs (1, 2). Although their inherent plasticity allows them to develop into various types and subtypes, three major types of blood monocyte subpopulations are recognized: the classical monocyte or CD14++ CD16–, which expresses high level of the LPS receptor (CD14) but no expression of Fc receptor (CD16), and the non-classical monocyte or CD14+ CD16++ characterized by low level expression of CD14 but higher level of CD16 expression. The third is an intermediate between the two, which expresses high level CD14 but low level CD16 (CD14++ CD16+) and is postulated to serve as a transitory link in the maturation process of the classical monocyte into the activated, non-classical monocyte (1, 2). The various monocyte types have been shown to exhibit distinct phenotype and function. Upon activation with microbial antigens, the non-classical monocytes are activated to produce high amounts of pro-inflammatory cytokines including tumor necrosis factor and IL-12 and exhibit higher potency in antigen presentation.

One of the major functions of blood monocytes is to eliminate antibody- or complement-opsonized microbes through either phagocytosis or by binding directly to the pathogen via pattern-recognition receptors (PRRs) that recognize pathogen-associated molecular ligands (3). Emerging among these PRRs are cC1qR and gC1qR, which in addition to their primary ligand, C1q, can also independently recognize a vast array of plasma proteins as well as pathogen-associated molecular ligands (4, 5). Moreover, monocytes express on their surface an intact macromolecular C1 as well as C1-INH (6, 7) with the globular heads free to recognize antigens. However, despite the available body of evidence showing the presence of macromolecular C1 on the surface of circulating blood monocytes (7), not much is known about its physiologic function. Based on the available data, we postulate that C1q – within the C1 complex expressed on circulating monocytes – may serve not only as a molecular sensor of danger but also as a molecular guarantor of steady state. Thus, while in the steady state, C1q within the C1 complex would regulate early processes that maintain cells in the monocyte or monocyte-like lineage, (*innate immunity*); recognition of “danger” would impart a license that drives monocytes toward the DC lineage (*adaptive immunity*). Deficiency in C1q therefore would disrupt this equilibrium.

## Dendritic Cells and Autoimmune Diseases

Dendritic cells are a complex lineage of antigen presenting cells (APCs) that orchestrate a variety of immune responses (3, 8–14). Although B and T cells are known to be the mediators of acquired immunity, their function is under the control of DCs. DCs in various stages of maturity capture, process and present antigens, express lymphocyte co-stimulatory molecules when activated, migrate to lymphoid organs, and secrete cytokines to initiate immune response (9). While B cells, the precursors of antibody-secreting cells, can directly recognize native antigen through their B-cell receptors, T lymphocytes need the antigen to be processed and presented to them by APCs such as DCs. The T cell antigen-receptors (TCRs) recognize fragments of the processed antigens bound to MHC molecules on the surface of DCs (9). The peptide-binding molecules on the APC are of two types: MHC class I (MHC I), which stimulates cytotoxic CD8+ T cells, and MHC class II (MHC II), which stimulates helper CD4+ T cells (8). A second co-stimulatory signal that is critical for T cell activation is the interaction of CD28 on the T cell and CD80/CD86 on the APC such as DC. In addition to their ability to activate lymphocytes, DCs can also tolerize T cells to self-antigens by a variety of mechanisms including the production of regulatory cytokines such as IL-10 and the induction of regulatory T cells (11, 14).

Dendritic cell precursors circulate in the bloodstream as monocytes, which are continuously generated from bone marrow progenitors. Migration into non-lymphoid organs induces differentiation of DC precursors into DCs that become resident tissue cells of the interstitium of peripheral organs or skin (9). These tissue-resident DCs are thought to be in an immature state [immature DCs (iDCs)] and are specifically characterized by high phagocytic activity and the ability to capture self and foreign antigens. The presence of specific lectins, such as DC-SIGN, Langerin, and mannose receptors on their surface allows iDCs to recognize invading bacteria or viruses (10, 13). Moreover, the expression of molecules such as α_v_β_5_ integrin and CD91 enables them to recognize and engulf self-antigens including those associated with apoptotic cells (10). Therefore, imDCs can interact with self-components, and virtually every antigen present in the periphery can be processed after engulfment and presented as peptide–MHC complexes on the DC surface (8, 11). Fortunately, tolerogenic mechanisms exist to prevent inappropriate autoimmune responses. Presumably, under *steady state* conditions DCs remain immature and start migrating towards lymph nodes upon partial activation signaling. Once they have reached the T cell area, these semi-mature DCs may induce tolerance by numerous mechanisms (3, 11, 14). In contrast, when DCs encounter a peripheral microenvironment characterized by pro-inflammatory factors and antigenic material, massive migration and maturation is triggered by molecules such as LPS, bacterial DNA, and double stranded RNA, which are recognized by specific Toll-like receptors (3, 12). Cytokines such as TNF-α and IL-1β found in the inflammatory compartment are also important participants in the DC maturation/activation process (9). The maturation of DCs is associated with the up-regulation of co-stimulatory molecules such as CD40, CD80, CD86, and CD58, secretion of cytokines, such as TNF-α, IL-6, and IL-12p70, the loss of endocytic phagocytic receptors, high levels of MHC I, II, CD83, and acquisition of high cellular mobility (13). The mature DCs migrate to the T cell areas of local lymph nodes where they are retained via specific chemokine interactions involving CCR7. Subsequent screening events result in engagement of the appropriately matched T cell-MHCI/MHCII receptors. Priming of naïve CD4+ T cells takes place when DCs engage CD40L. Because of their unique ability to initiate immune responses against invading pathogens as well as against peptides derived from self-proteins, DCs play an important role in the development of autoimmune diseases. However, a variety of mechanisms impact on the immunogenicity of DC in order to prevent autoimmune responses including *local factors* that facilitate *decisions* about the nature and subset of T cell response (15–18). The various signals that influence the DC are not yet fully elucidated, but these signals are likely to depend on the type and dose of antigen, the microenvironment of the DC–antigen encounter, the number, subset, and phenotype of the DC involved, the microenvironment of the secondary lymphoid organs where the antigen is presented, and finally the *local synthesis* of modulatory proteins including *complement proteins*, such as C1q, and its C1q receptors each of which is capable of recognizing and capturing self or non-self antigens.

## Expression of C1 and C1q Receptors on Monocytes and Dendritic Cells

**Figure 1:**
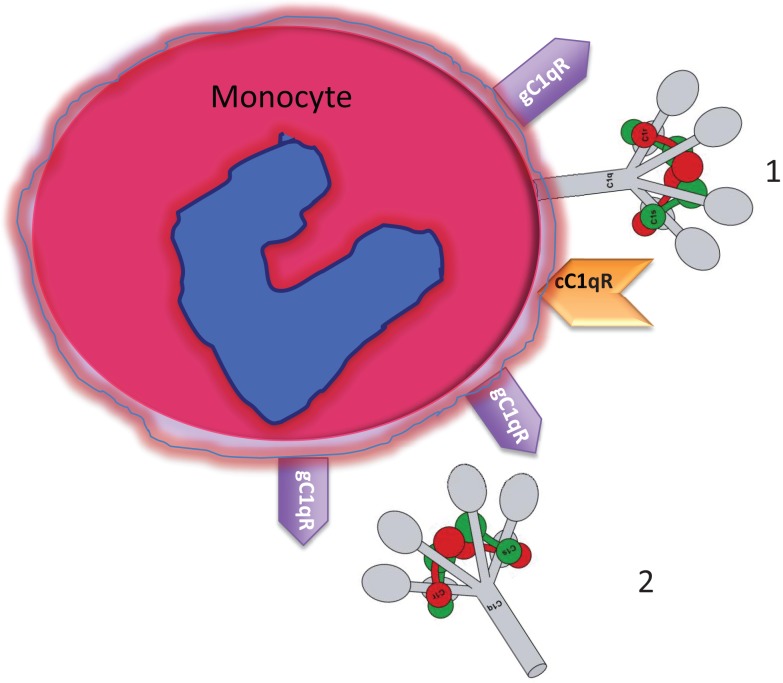
**Circulating blood monocytes express C1 and C1qRs**. (1) Membrane anchored C1q displays its globular heads free to interact with antigens or immune complexes. (2) Some of the membrane anchored C1q is cleaved off and secreted into the pericellular milieu and is able to bind to the cell surface via gC1qR. The C1q figure was obtained from Gérard Arlaud.

The synthesis of the subunits of the C1 complex (C1q, C1s, C1r), and its regulator C1 inhibitor (C1-INH) by human monocytes has been shown independently by several investigators (6, 19–24). However, the expression of the *intact* macromolecular C1 on the *cell surface* of monocytes and DCs has been described only recently (7). The first indication of the existence of a membrane form of C1 was intimated by the finding that monocyte-derived macrophages were able to synthesize and express a membrane anchored form of C1q (25). Furthermore, these original experiments showed that: (i) the surface expressed C1q was tightly and irreversibly anchored into the cell surface membrane with its globular heads displayed outwardly as evidenced by the fact that it was able to bind Fc, polyanions, the lipid A part of LPS, Gram-negative bacteria such as *S. minnesota*, as well as porins (outer membrane bacterial proteins); (ii) the A-chain of C1q contains amino acid sequences with properties characteristic of an integral type II membrane protein; and (iii) the surface molecule could only be liberated by detergent or repeated freeze-thawing (26, 27). However, these observations presumed that macrophages and *not their* monocyte precursors were able to express C1q on their surface. Using more sensitive assays and antibodies, recent experiments from our laboratory have shown that non-stimulated circulating blood monocytes are also able to both synthesize and express all of the components of C1 (C1q, C1r, C1s) as well as C1-INH (7). The C1 complex is presumably assembled around the C1q molecule in a manner that is similar to the C1 complex in plasma, with the C1q molecule serving as an anchored backbone to which the C1r_2_⋅C1s_2_ tetramer is linked via Ca^2+^ ions. Such a configuration, which is similar to the natural configuration of plasma C1 (Figure 1) would allow the exposure of the globular heads for immune complex binding or antigen recognition and complement activation. This assumption is also corroborated by the finding that the U937 expressed C1, is able to activate complement (7), and this activation is closely controlled by C1-INH, present on or near the cell surface (6). Collectively, the data suggest that the surface-associated C1 complex can initiate complement activation in response to an extracellular Ag and thus may represent the earliest response to either pathogen-associated or modified self-associated danger signals in blood.

**Figure 2:**
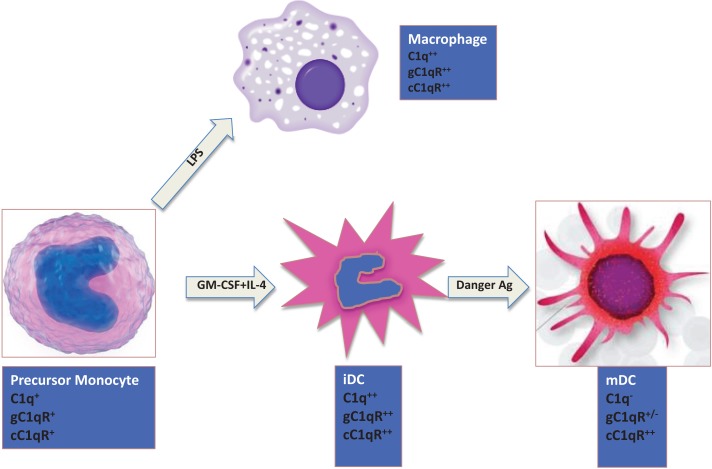
**Expression of C1q and C1qRs is maturation dependent**. Circulating monocytes express both C1 and C1qRs, which increase during the monocyte to iDC transition in the presence of self- or foreign-danger signals. Fully mature DCs, however, express little or no C1q, low gC1qR but high cC1qR.

The expression of C1q on monocytes is dependent on the maturation stage (Figure 2). While activated monocytes (macrophages) and immature DCs express elevated levels of C1q, this ability is lost when iDCs transition into mature DCs (mDCs) (28, 29). Interestingly, this expression profile is also mimicked by both C1q receptors (cC1qR and gC1qR), which in turn are co-localized with C1q and DC-SIGN on iDCs suggesting that C1q/gC1qR may regulate DC differentiation and function through the DC-SIGN-mediated induction of cell-signaling pathways (30). While inflammatory cytokines and LPS, which induce maturation of DCs, downregulate surface expression of both C1qR molecules, cytokines, and drugs such as IL-10, TNFα, or dexamethasone, that keep DCs phenotypically and functionally immature significantly upregulate the expression of both C1qRs (28).

The significance of C1q in the development of autoimmune diseases such as systemic lupus erythematosus (SLE) has been known for many years (31–33), and its potential role in tolerance induction has been proposed (34, 35). However, neither the mechanism by which C1q determines the activation thresholds of B and T cells nor how C1q deficiency causes incomplete maintenance of peripheral tolerance is clearly understood. The fact that C1q is co-localized with a number of surface molecules such as DC-SIGN, gC1qR, and cC1qR-implies that these molecules may collaborate in antigen recognition and processing (30).

## C1 Deficiency and SLE

Systemic lupus erythematosus – a prototype of a systemic autoimmune disease – affects close to three-quarters of a million individuals in the US and a much higher number worldwide with a frequency that varies by race and ethnicity with higher rates reported among Black and Hispanic people (36). Although it is a multifactorial disease, there is an overwhelming clinical evidence showing that homozygous deficiency in any of the classical pathway proteins – C1q, C1r, C1s, C4, and C2 – predisposes an individual to develop SLE and other autoimmune diseases such as rheumatoid arthritis (RA) (31, 33, 37). Among these proteins, C1q takes a prominent stage in significance as homozygous deficiency or hereditary deficiency due to mutation in the C1q gene is regarded as a strong susceptibility factor for the development of SLE (31, 33). The majority ( ≥95%) of the known individuals with C1q deficiency are known to have developed clinical syndromes closely related to SLE (31, 33, 38, 39). Interestingly, the majority of the circulating antibodies in SLE are against “modified self” or intracellular proteins (nuclear or cytoplasmic), suggesting that C1q may play an important role in the regulation and processing of these antigens.

**Figure 3:**
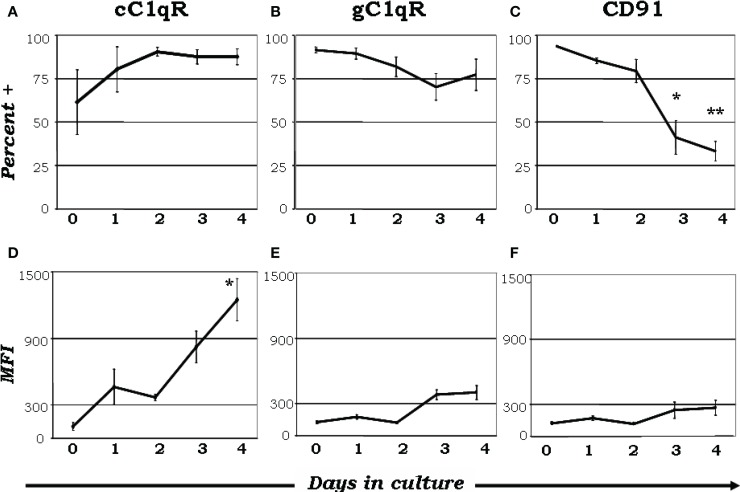
**Maturation dependent expression of C1qRs**. C1q receptors show varied expression on mono-DC precursors. Mononuclear cells were cultured in the presence of GM-CSF + IL-4, and collected and analyzed on days 0–4 for the expression of cC1qR **(A,D)**, gC1qR **(B,E)**, and CD91 **(C,F)** for both percent expression **(A–C)** and MFI **(D–F)**. **(A)** The percent of cC1qR expression was variable on monocytes, but by day 2 nearly all monocyte-DCs had the receptor on their surface. **(B)** On day 0, gC1qR was present on almost all the cells, and its expression was only slightly reduced by day 4. **(C)** Monocytes expressed CD91 on their surface, but the percentage of CD91+ cells was significantly reduced by day 3 and 4. **(D)** Mean fluorescence analysis revealed that cC1qR expression was dramatically amplified by day 3 and 4. **(E,F)** gC1qR and CD91 MFIs remained at relatively steady levels throughout the days. Experiments were gated on DR+ cells, *p < 0.05, **p < 0.01 (*n* ≥ 4) [adapted from Ref. (35)].

There has been an exponential increase in recent years exploring the possibility that C1q is a major link between *innate and acquired immunity*. Some of these studies have been made possible in part due to the availability of C1q^−/−^ animal models (40). As an archetypal pattern-recognition molecule with the ability to sense a wide variety of targets, C1q can engage a broad range of ligands – ranging from pathogen-associated molecular ligands (non-self) to damage-associated molecular targets (altered self) – including “eat me” signals such as self DNA and phosphatidylserine, which are the first structures exposed at the apoptotic cell surface (41–45). These interactions are able to trigger a multiplicity of immunologic functions, which by and large are beneficial to the host. It is not therefore surprising that deficiency in C1q leads to various diseases including otitis media, meningitis, pneumonia (31, 34, 37) as well as autoimmune diseases, such as SLE (31, 46, 47). Several reports have shown that C1q can bind via its globular “heads” to the surface of apoptotic cells (44, 47); and the common autoantigens targeted in SLE are found in high concentrations on the surface of apoptotic cells (44, 47). However, although it is clear that C1q and its receptors play a role in removal of self-waste (44, 47–50) the existence of functionally redundant pathways for apoptotic body clearance gives credence to the postulate that autoimmunity arising from diminished C1q activity could reflect another role of C1q in maintaining tolerance (34). Thus, while C1q could provide active protection from autoimmunity by silencing key molecular markers or regulating autoreactive immune cells, its absence or defective expression could lead to a loss of peripheral tolerance as a cumulative result of impaired apoptotic cell clearance in conjunction with negative signaling. For example, incubation of C1q with T cells has been shown to inhibit T cell proliferation presumably by binding to gC1qR on the T cell surface (51) since this activity is mimicked by incubation of cells with mAb 60.11, which recognizes the C1q binding site on gC1qR. Furthermore, although its role is not precisely known, soluble gC1qR is able to bind CD4 thereby blocking HIV-1 viral entry into T cells (52). More importantly, because of its heterotrimeric nature and oligomeric structure – with two distinct structural and functional domains (gC1q and cC1q) – C1q is also able to interact with cell surface receptors via either its cC1q or gC1q domains. Similar to C1q, the C1q receptors are differentially expressed as monocytes go through the maturation process to become mDCs (35). When monocytes are cultured in the presence or absence of GM-CSF + IL-4, nearly all monocyte-derived DCs expressed gC1qR on day 0 (Figure 3B), while cC1qR was more variable within the population (Figure 3A), despite the consistently elevated expression of its putative surface partner, CD91 (Figure 3C). Even though there was a modest reduction in gC1qR^+^ cells by day 4 (Figure 3B), the percent of cC1qR expression increased compared to day 0 (Figure 3A). Furthermore, mean fluorescence index (MFI) analysis revealed that the amount of cC1qR was dramatically amplified after day 2 (Figure 3D), whereas the amount of gC1qR remained at relatively steady levels (Figure 3E). Thus, at the precise period (~day 3) corresponding to firm commitment to the DC lineage there is an inverse correlation between gC1qR and cC1qR expression on the cell surface, which in turn may influence the nature and specificity of the cells’ response to C1q. Interestingly, despite the increase in cC1qR expression, its surface partner, CD91, was gradually reduced during the culture period, indicating that alternate partners for cC1qR are present upon commitment to the DC lineage (Figures 3C,F). Taken together, these data suggest that the regulatory effects of C1q on DC differentiation and function may depend on specific C1q/C1qR interactions; and these interactions may in turn control the transition from the monocyte state (*innate immunity*) toward the professional APC state (*adaptive immunity*). The observation that soluble C1q functions as a “*molecular switch*” during the narrow window – i.e., at the precise period (~day 3) corresponding to firm commitment to the DC lineage – of monocyte to DC transition (35) not only explains why C1q is primarily synthesized, expressed, and secreted by potent APCs, but also why its absence can impair antigen uptake and tolerance.

**Figure 4:**
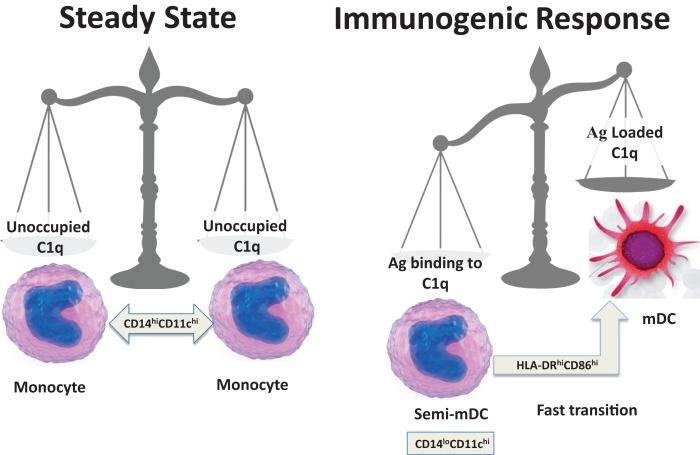
**Schematic representation of a fine-tuned balancing act**. The transition from steady state to immunogenic response depends on the encounter between an antigen and C1q.

On the basis of the available data and our own recent findings (35), we speculate that soluble C1q induces inflammatory responses by binding through its gC1q to pathogen-associated molecular patterns (PAMPs) and to modified self antigens (DAMPs), and stimulates phagocytic cells through interactions of its cC1q domain with cC1qR. According to this *hypothesis*, a normal response to “*danger*” would involve up-regulation of cC1qR on iDC to ensure uptake of noxious agents utilizing the Ag-retrieving functions of C1q (44, 48). In the context of inflammatory stimuli, DC maturation would ensue allowing adaptive immune responses against the initiating agent. In contrast, C1q that is free of antigenic cargo (i.e., in the absence of *danger*) does not support full commitment to the DC lineage, and instead keeps them in a monocytic phenotype as represented during steady state. Therefore, during normal physiology, return to steady state levels of C1q/C1qR on monocytes and/or DC precursors would resume once pathogen/danger has been cleared (Figure 4).

Similar to the suppressive effect of C1q on T cells acting through gC1qR/C1q interactions (51, 53), we hypothesize that the regulatory effects of C1q on monocyte/DC precursors may occur via engagement of gC1q. Due to the swift nature of the monocyte to DC transition, regulatory effects of a C1q/C1qR system would occur within a narrow time frame and would be influenced by the microenvironment (steady state or infection or inflammation). The dichotomy of the two apparently opposing roles of C1q in turn are a result of the binding orientation of C1q – *heads* versus *tails* – and the specific receptors engaged (*gC1qR* versus *cC1qR*) on the cell surface. Such duality of function would be very similar to the role of surfactant proteins (Sp)-A and Sp-D in the lung, which help maintain the steady state environment via binding to the ITIM-containing SIRPα through their globular head domains or initiate ingestion and pro-inflammatory responses through the collagenous tails and cC1qR (calreticulin)/CD91 (54). Furthermore, although the significance of these motifs has yet to be elucidated, another intriguing observation in this context is the presence of dueling ITAM/ITIM motifs around the tyrosines at positions 224 and 236, respectively on gC1qR, which is unique for a molecule that is both an intracellular protein and a cell surface receptor (55). The observations described above together with the finding that there is a clear differential pattern of C1qR expression during the monocyte to DC transition (28, 35), allows us to hypothesize that preferential engagement of distinct regions of C1q (globular heads versus collagen tails) takes place during different stages of DC growth. Accordingly, the globular head domain of the molecule would mediate regulatory effects of C1q. Since the collagen tail of the C1q in plasma or on the cell surface would be occupied, this association only allows the C1q subunit to bind gC1qR and not cC1qR on the cell surface. In support, we have found that some or all of the C1q detected on the monocyte surface is part of the C1 complex (7). Furthermore, cC1qR has been shown to bind C1q only when it undergoes a conformational change (e.g., through immobilization on a surface) (56). Such altered conformation may take place when C1q binds antigen or immune complexes.

## Effect of C1q on DC Differentiation and Function

The development of monocyte-derived DCs from human peripheral blood (PB) is marked by the rapid loss of CD14 (receptor for LPS), up-regulation of CD11c [integrin, α_x_ (complement component 3 receptor 4 subunit (CR4)], HLA-DR (MHC class II), and co-stimulatory and maturation associated molecules (such as CD86, CD80, CD83) (57–59). Distinct subsets of DC precursor populations have been described to arise from human PB monocytes treated with GM-CSF + IL-4. These include CD14^−^CD11c^+^, CD14^−^CD11c^−^, and CD16^±^CD14^+^CD11c^+^ cells (58, 59). While the majority of iDCs avidly ingest potentially antigenic material as a prerequisite for an immunogenic response, failure to undergo terminal maturation into APCs results in a potentially tolerogenic state (8, 11). Targeting of various uptake receptors, notably of C-type lectin receptors (e.g. DC-SIGN, Langerin, BDCA-2, Dectin-2, etc.) by self-derived Ag on iDCs leads to tolerance by default (8, 11), whereas pathogen-derived Ag might simultaneously signal through TLRs and C-type lectins, thus induce immunity (8, 11).

**Figure 5:**
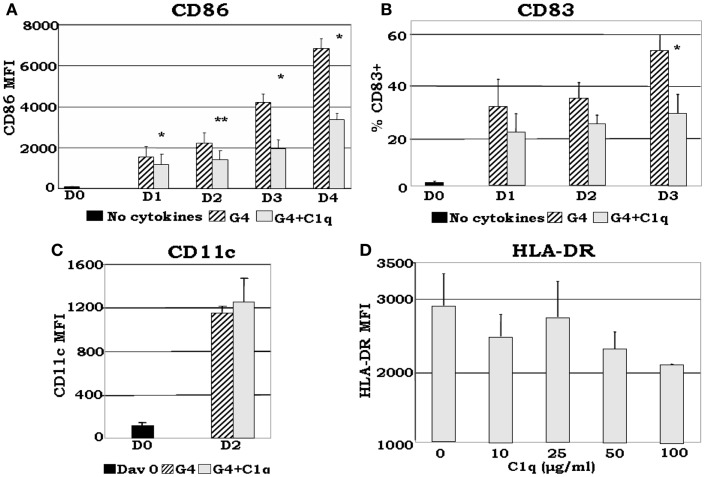
**C1q delays GM-CSF + IL-4 induced DC maturation**. Monocyte-DCs were isolated and cultured in the presence of GM-CSF + IL-4 (G4) and with or without 25 μg/ml C1q **(A–C)**. For the dose response experiments several concentrations of C1q were added as indicated **(D)**. Cells were collected on the days indicated and analyzed for the expression of CD86, CD83, and CD11c for each condition **(A–C)**, or alternatively, on day 2 HLA-DR analyses were performed for all C1q doses **(D)**. **(A)** C1q significantly decreased CD86 expression in monocyte-DCs compared to G4, *p < 0.05, **p < 0.01 (*n* = 4); **(B)** monocyte-DCs cultured in the presence of C1q showed a decrease in the percentage of CD83+ cells in comparison with cells cultured in G4 alone. While CD83 expression was detected on the surface of these cells, their MFI remained low throughout the days with or without the addition of C1q (data not shown), *p < 0.05 (*n* = 4); **(C)** CD11c expression was increased by day 2 with the addition of C1q compared to day 0. There was no significant difference in CD11c expression levels on cells cultured with or without C1q (*n* = 6); **(D)** dose response analysis revealed that the MFI of HLA-DR+ cells correlates negatively with increasing doses of C1q on day 2. While there was little or no difference in the distribution of HLA-DR on the cells at lower doses (10–25 μg/ml), at higher doses decreased expression was noted (MFI) (*n* = 3). Cells were gated on DR+ cells for all experiments [adapted from Ref. (35)].

Although somewhat contradictory to the findings of others, which showed that C1q induces maturation of DCs (60), recent data from our laboratory (35) show that: (i) even in the presence of DC growth factors, exogenously added C1q is able to inhibit the expression of monocyte-derived DC maturation markers such as CD86, a required co-stimulatory molecule for T cell activation (Figures 5A,B); (ii) C1q promotes the development of distinct iDC subsets CD14^hi^CD11c^hi^CD16^±^HLA-DR^hi^CD86^dim^ (Figures 5C,D) compared to CD14^−^CD11c^hi^CD16^−^HLA-DR^hi^CD86^hi^ cells, which develop without C1q. The decreased expression of maturation dependent markers on C1q treated versus untreated cells substantiates the notion that C1q alters monocyte to DC differentiation. In addition to these observations, our previously published results (7, 35) indicate that Ag-free, extracellular C1q exerts a regulatory signaling function through engagement of the globular heads by surface gC1qR and thus modulate the differentiation of monocyte-DC precursors and induce the development of CD14^hi^CD11c^hi^CD16^+/−^cells. Therefore, the decreased expression of DC maturation dependent markers and the increased expression of monocyte associated surface molecules in the presence of C1q support the concept that the developing cells acquire features of both monocyte and DC. Because C1q is structurally similar to SP-A and SP-D, it might also perform a *dual* function in that it may help maintain the steady state environment via binding to ITIM-containing surface molecules on cells (54, 61) including gC1qR through its globular head domains. Accordingly, occupancy of the gC1q by foreign Ag would induce a conformational change (56) thereby making the collagen tail available for binding to cC1qR. The ensuing cell differentiation and signaling events would thus support rapid DC maturation and immune activation. Conversely, self-derived Ag bound C1q might relay signals through C1q or C-type lectin receptors only, thus inducing a strong tolerogenic response, resulting in development of tolerogenic DC and Treg activation.

## Insights in to the Mechanisms of C1q-Mediated Monocyte/DC Signaling

To date, the information available on the signaling events regulated by C1q in DCs is very limited. Some studies using murine bone marrow-derived DCs, have shown that C1q treatment suppresses IL-12 production, and reduces the phosphorylation of p38, ERK1/2, c-Jun N-terminal kinase, and extracellular signal-regulated kinase after stimulating the cells with LPS, CpG oligodeoxynucleotides, or anti-CD40 antibodies (62, 63). This is contrary to the findings by others that C1q induces maturation of DCs and secretion of IL-12 and TNF-α and elevated their T cell stimulating capacity (60). However, a definitive mechanism that mediates C1q signaling in human DCs has not been described. Understanding the molecular mechanisms of how C1q regulates adaptive immune functions via iDCs in the absence of infection or inflammation is therefore highly significant. Since our data indicate that CD14 levels are markedly increased on mono-DCs when they are cultured in the presence of C1q (35), signaling blockades that may potentially be mediated by CD14 should be evaluated. LPS triggers CD14/TLR4 mediated signaling to induce cell differentiation via TRAF6 and IRAK4 signaling. Although the effect of C1q on CD14^−^ and TLR-mediated signaling cascades in mono-DCs has not been yet fully investigated, ligand engagement of gC1qR (HCV core protein and mAb) has been shown to increase PI3K activation and Akt phosphorylation in LPS stimulated monocytes in an ERK independent manner (64). Moreover, DC-SIGN, which forms a molecular complex with C1q and gC1qR on the surface of iDCs (30), has been shown to increase phosphorylation of Raf-1 on Ser338 and Tyr340/341 in a ligand-dependent manner (65). Furthermore, stimulation of DC-SIGN with the mannose receptor-1 (MR-1) Ab has been shown to induce activation of the MEK/ERK kinase cascade (66). Whether direct stimulation of C1q participates in these signaling pathways, however, still remains to be investigated.

## Concluding Remarks and Future Outlook

The present paper is intended to provoke a debate and inject novel insights into the endlessly intriguing question: *Why does homozygous deficiency in C1q predispose an individual to develop SLE and other autoimmune diseases?* Although the contribution of C1q to the process of apoptotic cell clearance is unquestionable – and is supported by robust experimental data – the presence of functionally redundant pathways for removal of apoptotic bodies together with the direct effect of C1q on monocytes/DCs and T cells would suggest that, in addition to its role in apoptotic clearance, locally secreted C1q – which is DC maturation dependent – determines the activation thresholds of B and T cells and that C1q deficiency causes incomplete maintenance of peripheral tolerance. Therefore, while in the steady state, C1q would regulate cells during the early DC differentiation events by silencing critical phagocytic or stimulatory markers requisite for antigen presentation (*innate immunity*), recognition of “danger” would, on the other hand, impart a signal that drives monocytes toward the DC lineage (*adaptive immunity*). Deficiency in C1q therefore will disrupt this equilibrium. In addition, in the absence of danger signals, C1q may help maintain steady state conditions by skewing DC differentiation toward a “hybrid” cell type with both monocyte–macrophage-like (increased CD14, enhanced phagocytosis, IFN-γ secretion) and DC-like (T cell priming) characteristics. The distinct binding orientation of C1q (heads versus tail) on the monocytes and iDCs also suggests that specific C1q/C1qR interactions – and possibly other surface molecules – may regulate cells as they transition from the monocyte state toward the professional APC state. The presence of C1q together with C1r, C1s, and C1-INH on the monocyte surface with its versatile antigen-capturing region displayed outwardly also supports the concept that monocytes may represent the first sentinels of danger in the blood.

However, although these postulates are conceptually appealing and are supported by experimental data, future studies, which focus on the role of C1qRs, C1q, and C1 within the microenvironment of the monocyte/DC–antigen encounter will be necessary if we are to gain insight into why deficiency in any of the components of C1 leads to the development of autoimmunity. More importantly, information derived from such studies will not only provide new groundwork for future undertakings related to innate and acquired immunity, but also would give credence to targeted modulation of C1/C1q-mediated DC function as new treatment option aiming at alleviating DC-driven autoimmune responses.

## Conflict of Interest Statement

The authors declare that the research was conducted in the absence of any commercial or financial relationships that could be construed as a potential conflict of interest.

## Abbreviations

cC1qR, receptor for the collagen-like region of C1q; DC, dendritic cell; gC1qR, receptor for the globular heads of C1q; iDC, immature DC; mDC, mature DC.
